# Supplementary material for: Kinesin-1 activity recorded in living cells with a precipitating dye
Source: Nat Commun. 2021 Mar 5;12:1463. doi: 10.1038/s41467-021-21626-1 (PMC7935933; doi:10.1038/s41467-021-21626-1)
Supplement: Supplementary file 1 — Supplementary Information [file 41467_2021_21626_MOESM1_ESM.pdf]

Supplementary Information

Kinesin-1 activity recorded in living cells with a precipitating dye

Simona Angerani[1], Eric Lindberg[1], Nikolai Klena[2], Christopher K. E. Bleck[3], Charlotte Aumeier[1]* and Nicolas Winssinger[1]*

1. School of chemistry and biochemistry, NCCR Chemical Biology, Faculty of Science, University of Geneva, 1205 Geneva, Switzerland
2. Department of cell biology, Faculty of Science, University of Geneva, 1205, Geneva, Switzerland
3. Electron Microscopy Core Facility, National Heart, Lung and Blood Institute, National Institutes of Health, Bethesda, MD, USA

Supplementary Figures...S2

Supplementary Methods...S13

Supplementary References...S17

Supplementary Figures

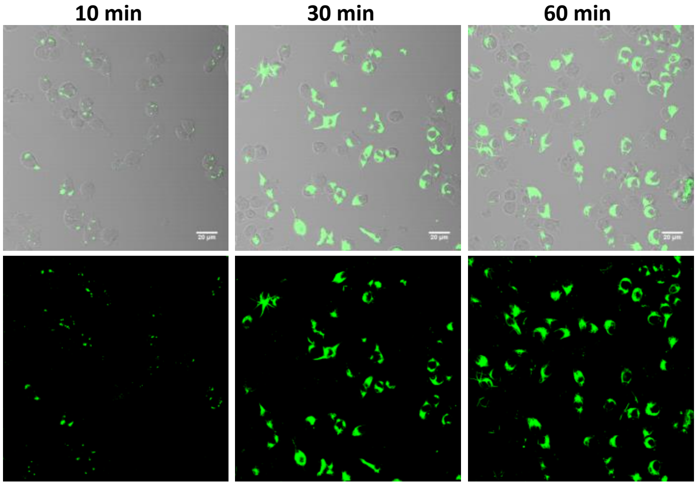

Supplementary Figure 1. QPD-OTf in RAW246.7 cells. QPD fluorescence (green) (bottom) at different time points and merge images (top). Scale bar 20 µM.

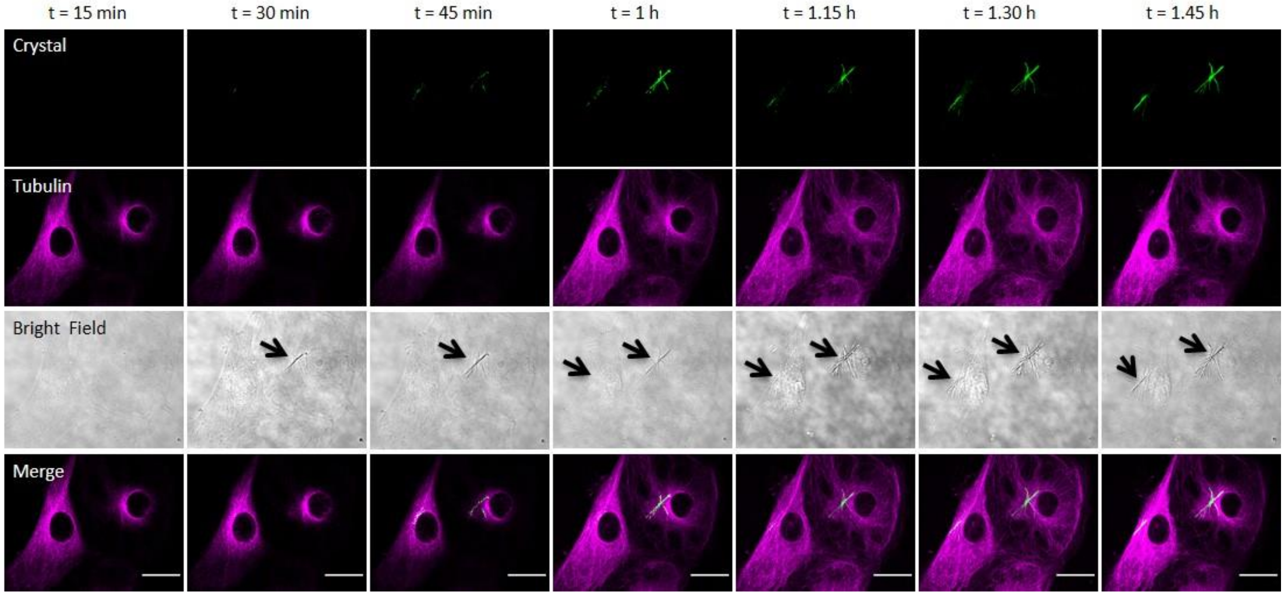

Supplementary Figure 2. **Crystal formation over time.** PTK2-GFP-Tubulin cells treated with QPD-OTf (20 µM) imaged over time. Green: QPD fluorescence; magenta: GFP-tubulin. Black arrows indicate the position of crystals. Scale bar 20 µM.

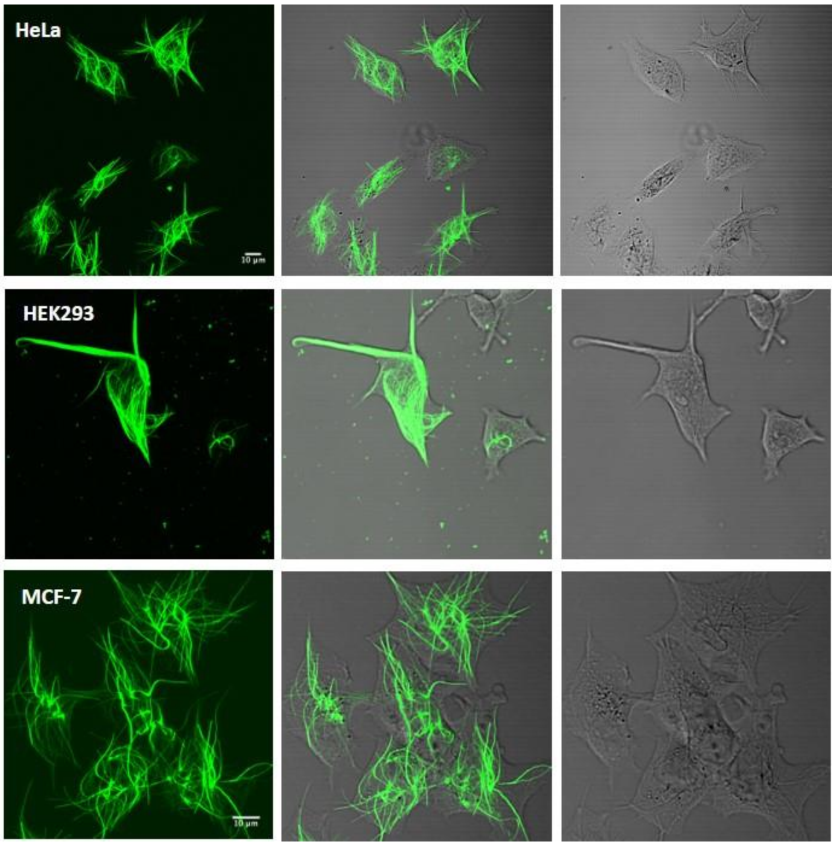

Supplementary Figure 3. **QPD-OTf in a panel of human cell lines.** QPD fluorescence (green) (left); merge (centre); bright field (right). Scale bar 10 µM.

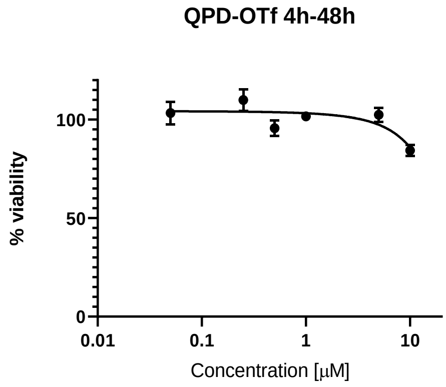

Supplementary Figure 4. WST-1 viability assay in MCF-7 cells. Cells were treated with QPD-OTf for 4 hours; washed, incubated for 48 hours, then WST-1 assay was performed. Experiments were performed in triplicates; curve fitting was performed with GraphPad 8; data are presented as mean value +/- the standard deviation (SD). Source data are provided as Source Data file.

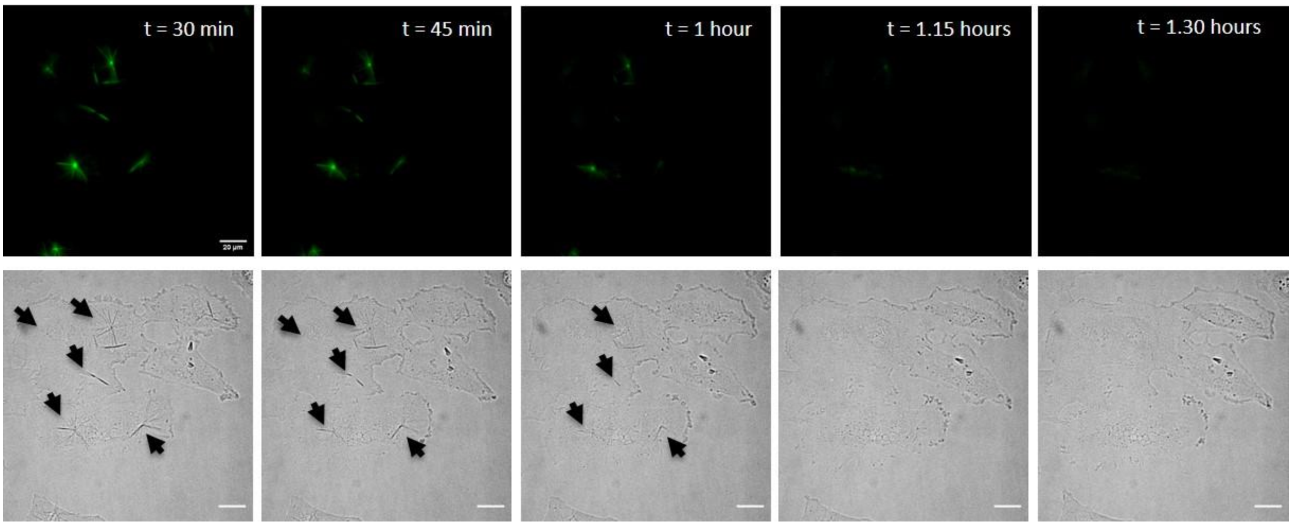

Supplementary Figure 5. Crystals wash out. HeLa cells treated with QPD-OTf (20 µM, 1.5 hours), washed and imaged over time to observe crystal disappearance. Green: crystals (top); bright field images (bottom); black arrows indicate the position of crystals. Labels refer to the time after wash out. Scale bar 20 µm.

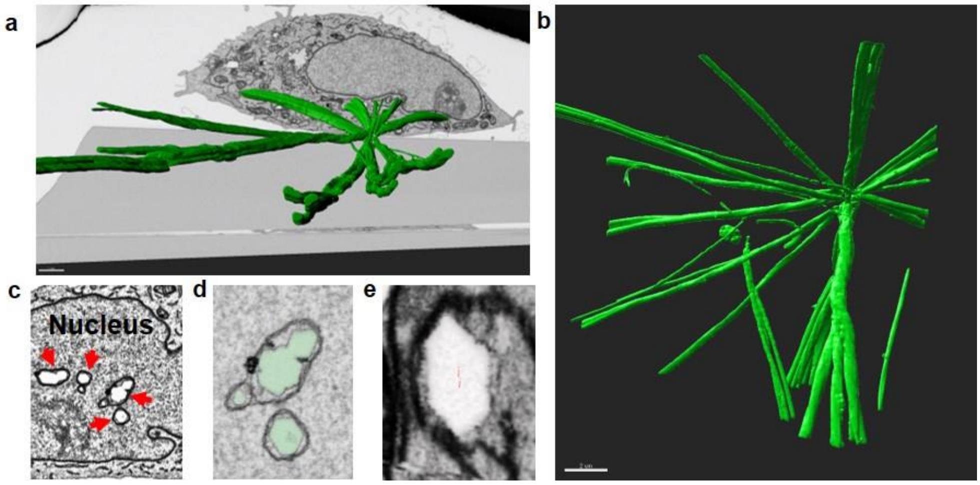

Supplementary Figure 6. 3D reconstruction of crystals in HeLa cells from FIB-SEM analysis. **a**) Crystal (green) over cell section. Scale bar 1 µM. **b**) Side view of crystal. Scale bar 2 µM. **c**) FIB-SEM cross section of HeLa cell containing crystals spanning through the nucleus (bottom); red arrows indicate the crystal sections. **d**), **e**) Cross sections of crystal fibers.

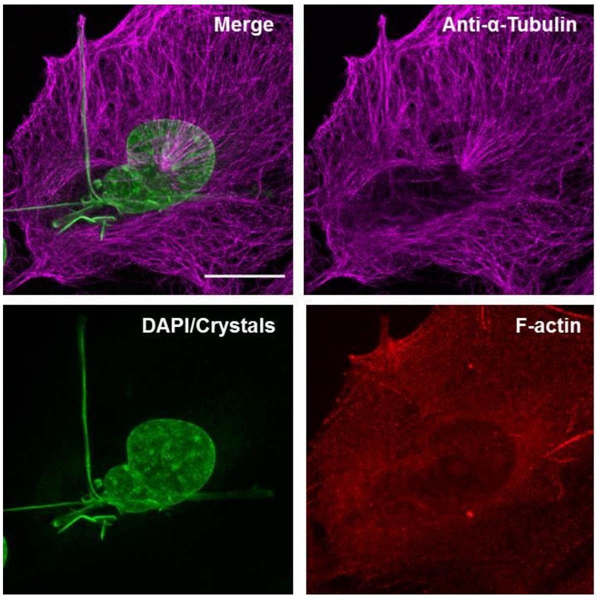

Supplementary Figure 7. Immunofluorescence of U2OS cells treated with QPD-OTf. Fixed U2OS treated with QPD-OTf and stained with DAPI, α-tubulin immunostaining and actin immunostaining. Green: DAPI/Crystals; magenta: anti-α-tubulin; red: F-actin. Scale bar 20 μM.

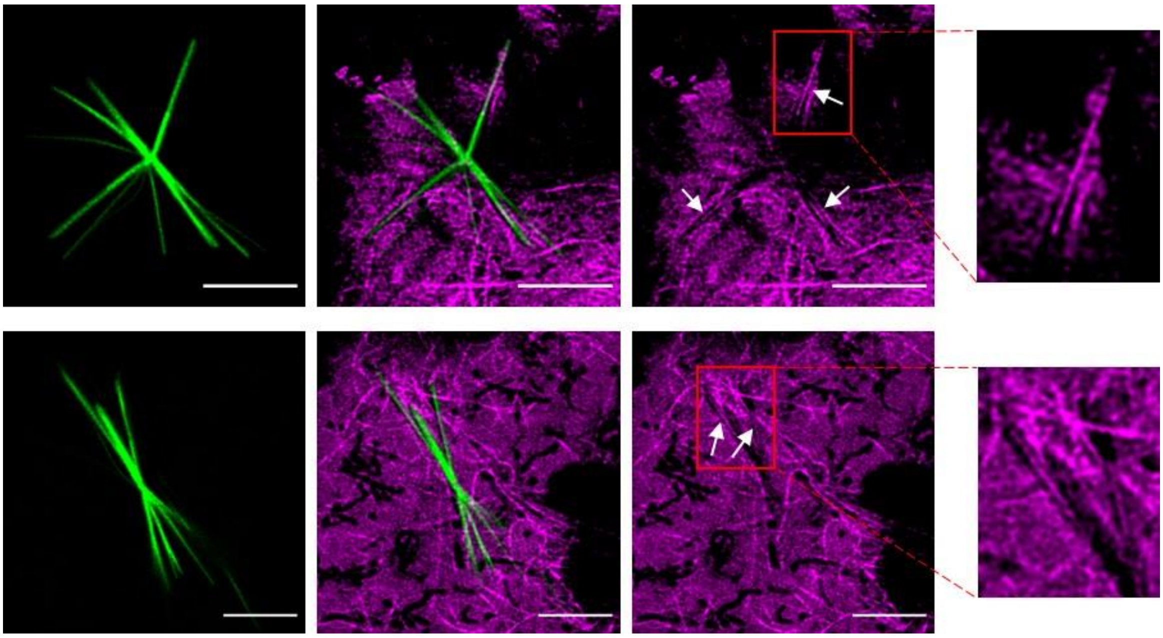

Supplementary Figure 8. Colocalization of crystals with MTs. Super-resolution images of HeLa-GFP-Tubulin treated with QPD-OTf (20 μM; 2 hours); white arrows indicate MTs colocalization with crystals; red boxes indicate the portion of image zoomed-in (GFP-Tubulin: magenta; crystal: green). Scale bar 5 μM.

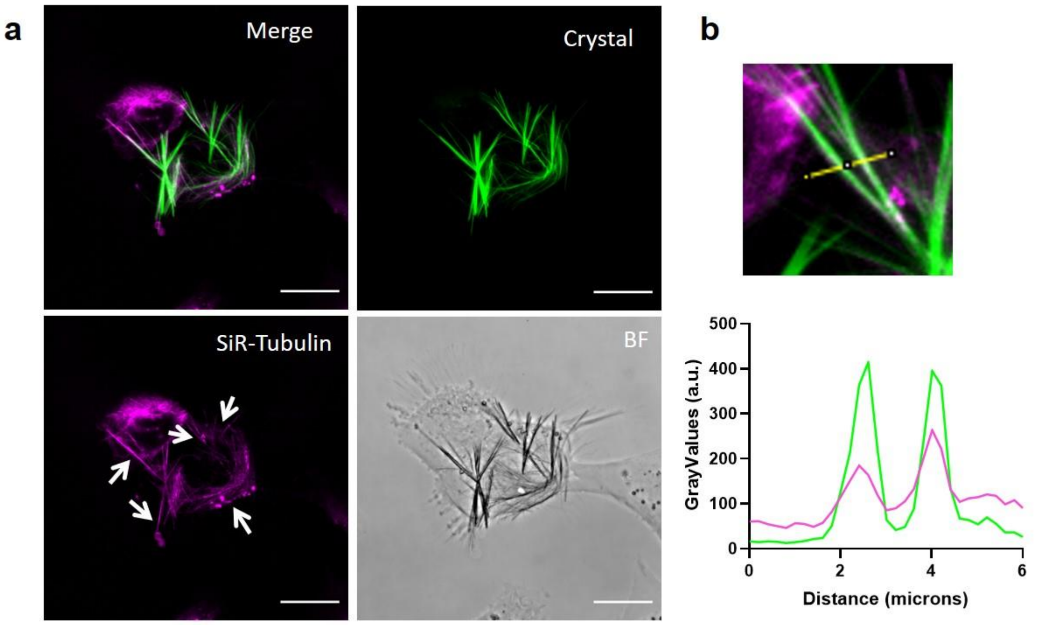

Supplementary Figure 9. Colocalization of crystals with SiRTubulin staining. a) Tubulin staining in live U2OS cells treated with QPD-OTf; white arrows indicate colocalization with crystals (SiR-Tubulin: magenta; crystal: green). **b**) Plot profiles of tubulin channel and QPD channel (SiR-Tubulin: magenta; crystal: green; yellow line represents the section plotted in the graph) (a.u. represent arbitrary units). Scale bar 20 µM. Source data are provided as Source Data file.

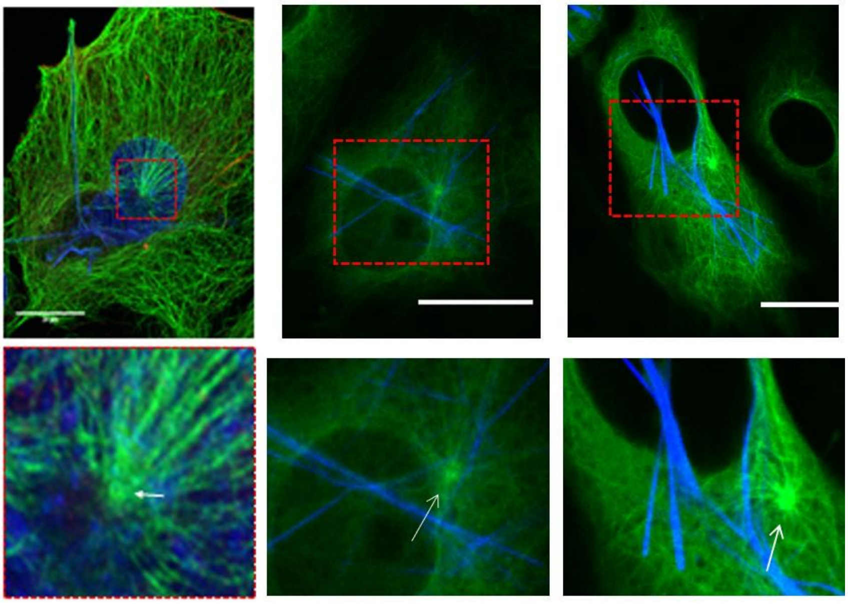

Supplementary Figure 10. The centrosome does not exhibit colocalization with the nucleating site for QPD precipitation. Representative images of U2OS or PTK2-GFP-Tubulin treated with QPD-OTf (2 hours, 20 µM). Green: tubulin; blue: crystal/DAPI. Arrows indicate the position of the centrosome. Red dotted-boxes indicate the zoomed-in area of the pictures shown in the bottom panels. Scale bar 20 µM.

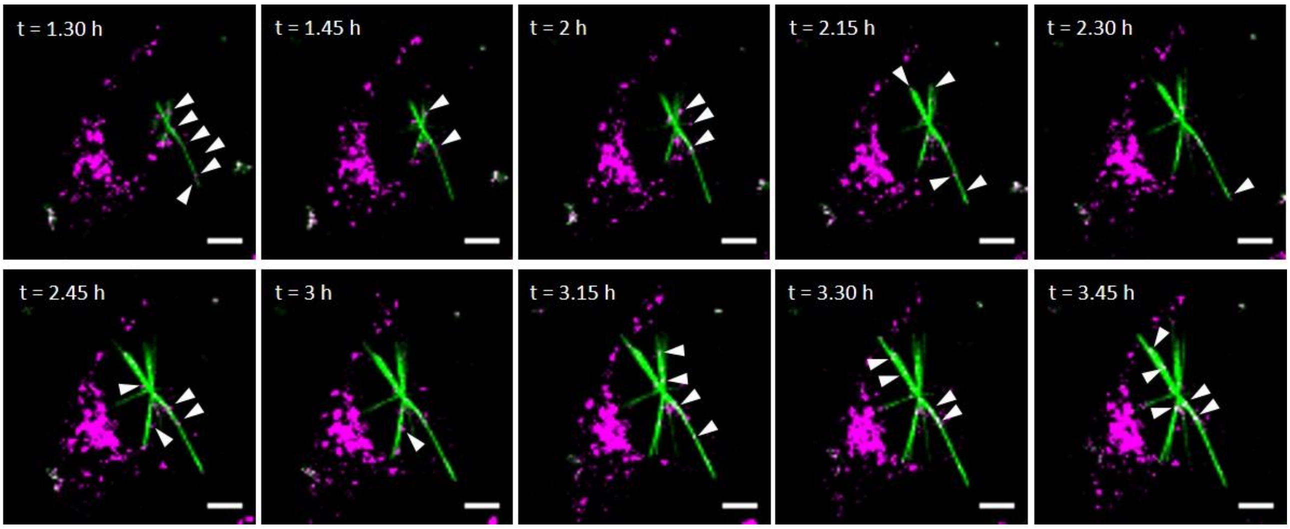

Supplementary Figure 11. Crystal growth in U2OS cell over in relation to Golgi vesicle transport. U2OS cells transfected with mCherry-Giantin plasmid and treated with QPD-OTf (20 μM). White arrowheads indicate Golgi vesicles along crystal filaments. Green: crystal; magenta: Golgi. Scale bar 5 μM.

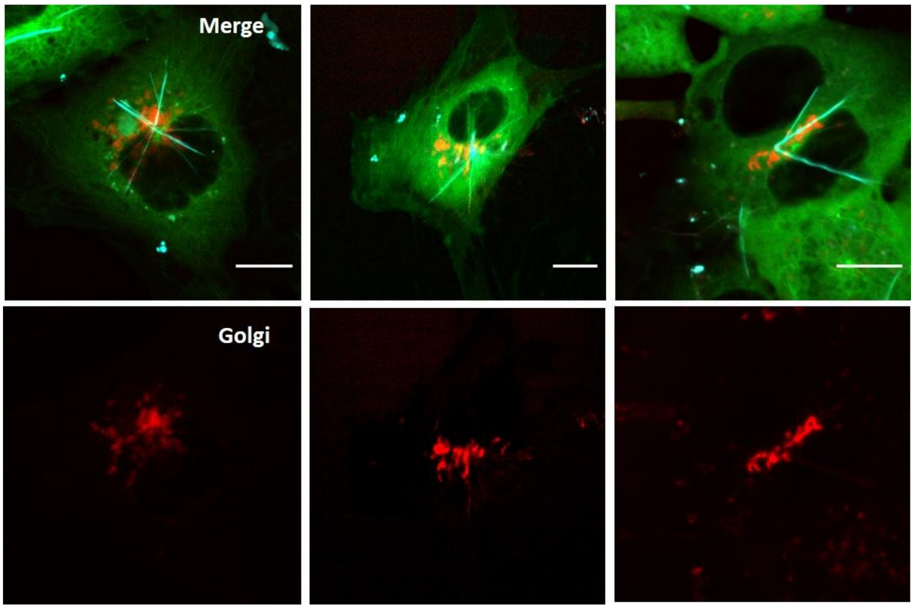

Supplementary Figure 12. Colocalization of the center of crystals with Golgi apparatus. PTK2-GFP-Tubulin cells were transfected with mCherry-Giantin plasmid and treated with QPD-OTf (20 μM, 2 hours). Green: tubulin; cyan: crystals; red: Golgi. Scale bar 10 μM.

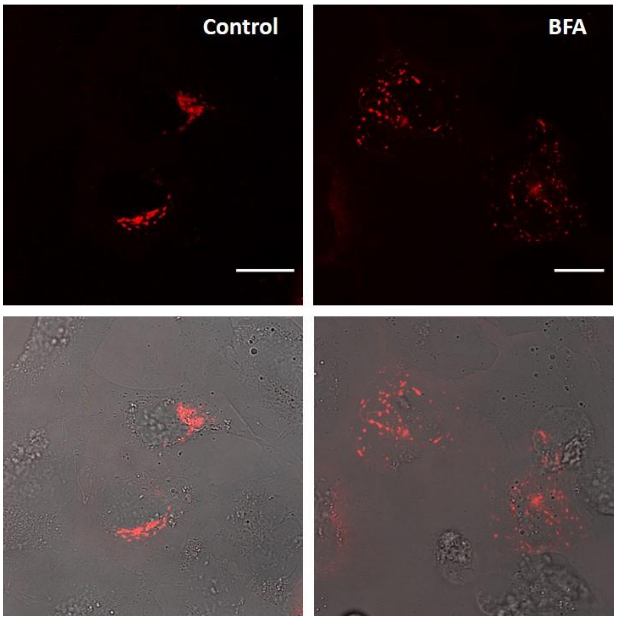

Supplementary Figure 13. Imaging of Golgi apparatus in cells treated with BFA. U2OS cells were transfected with mCherry-Giantin plasmid before (Control) and after (BFA) treatment with Brefeldin A (20 μM, 4 hours). Red: Golgi. Scale bar 20 μM.

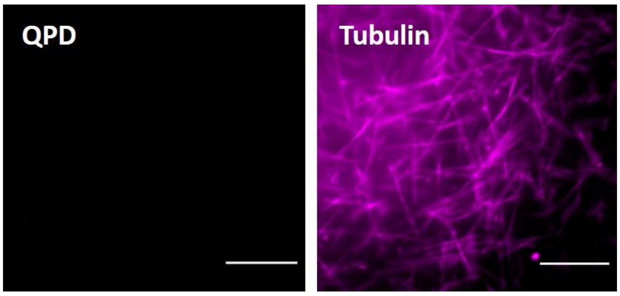

Supplementary Figure 14. QPD-OTf treated MTs in vitro. TIRF images of QPD-OTf treated MTs (20 μM, 2 hours). Magenta: tubulin. Scale bar 10 μM.

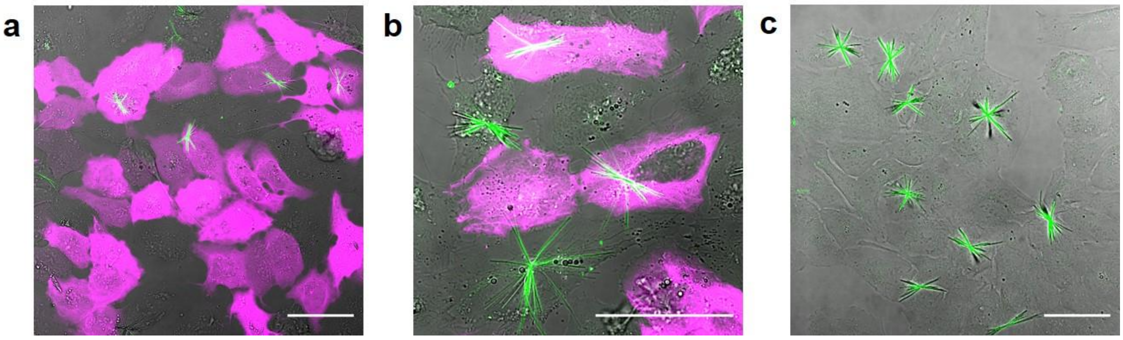

Supplementary Figure 15. Imaging of transfected truncated kinesin in QPD-OTf treated U2OS. a) Merge image of U2OS cells transfected with Kin330-GFP plasmid and treated with QPD-OTf (20 μM, 2.5 hours); green: crystals, magenta: kin330-GFP. **b**) Merge image of U2OS cells transfected with Kin560-GFP plasmid and treated with QPD-OTf (20 μM, 2.5 hours); green: crystals, magenta: Kin560-GFP. **c**) Control: U2OS cells treated with QPD-OTf (20 μM, 2.5 hours); green: crystals. Scale bar: 50 μm.

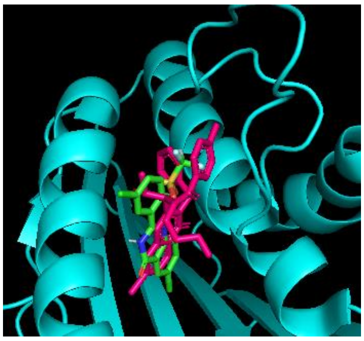

Supplementary Figure 16. **QPD-OTf docking into the Ispinesib binding site of Eg-5**. Superposition of Ispinesib (magenta) and QPD-OTf (green) into Eg5 protein (cyan).

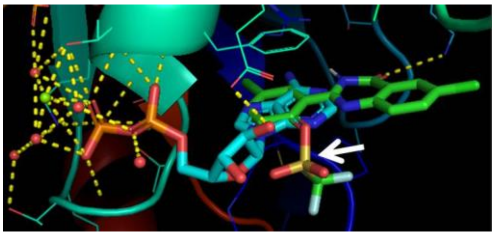

Supplementary Figure 17. QPD-OTf docking into the ATP binding site of Eg-5. Superposition of QPD-OTf (green) and ADP (cyan). White arrow indicates the triflate group of QPD-OTf.

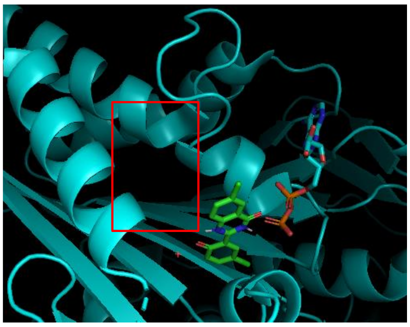

Supplementary Figure 18. QPD-OTf docking into the Ispinesib binding site of kinesin-1 co-crystallized with ADP. QPD-OTf (green), ADP (cyan backbone; orange phosphate groups) into kinesin-1 (cyan). Red box indicates the "Ispinesib-like" binding site.

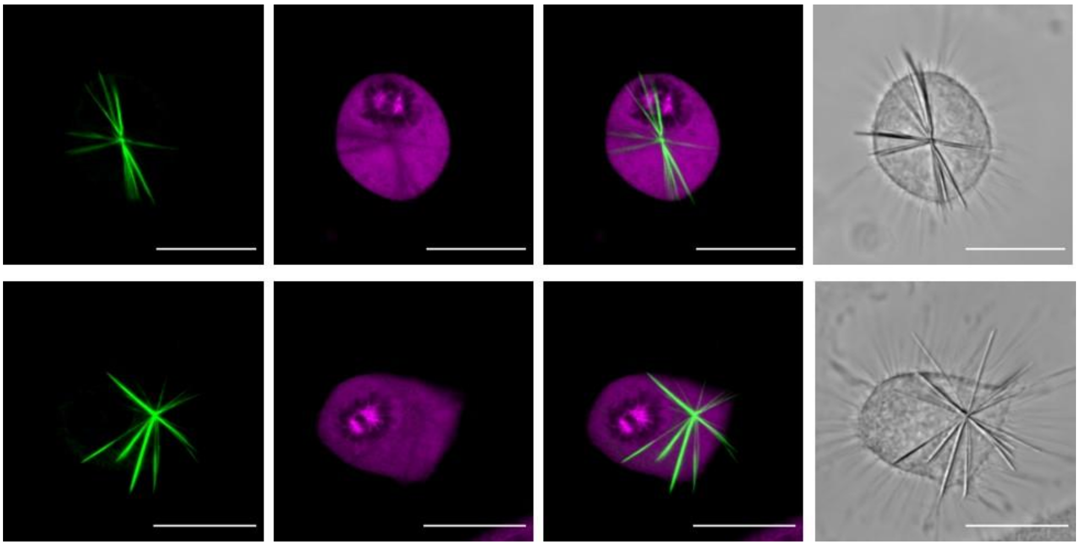

Supplementary Figure 19. Crystal location in mitotic cells. HeLa-GFP-Tubulin cells treated with QPD-OTf (20 µM). Green: crystal; magenta: tubulin. Scale bar 20 µm.

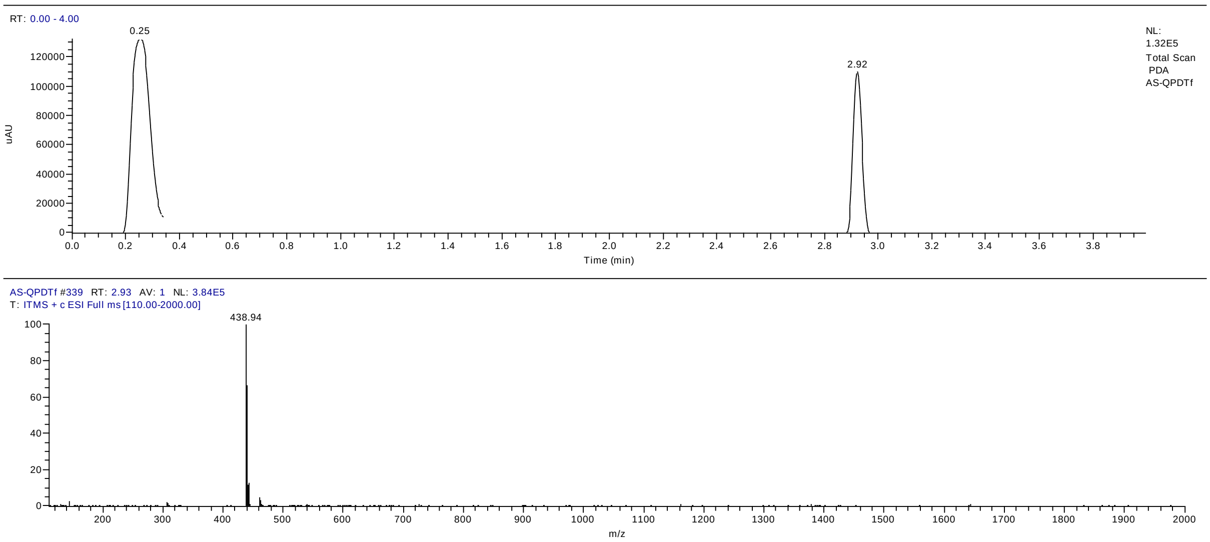

Supplementary Figure 20. LC-MS trace of QPD-OTf.

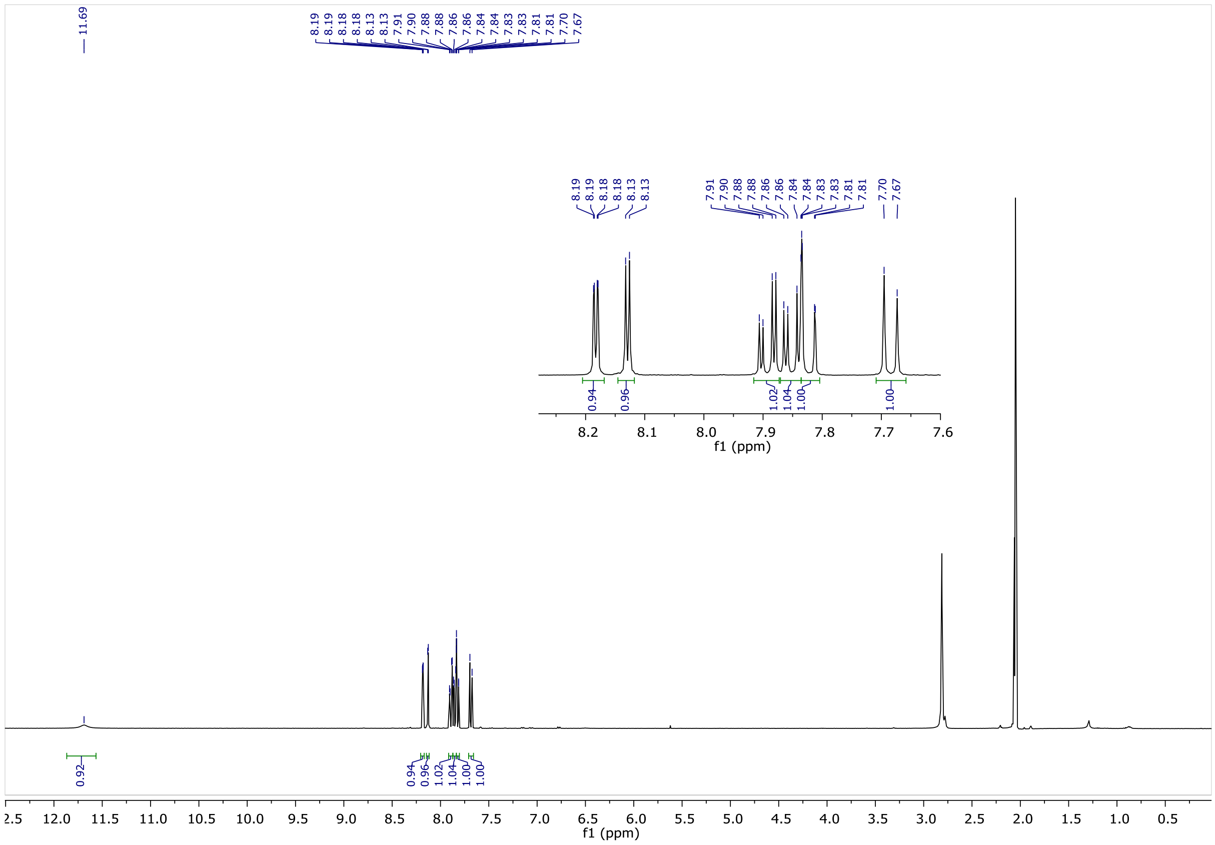

Supplementary Figure 21. ^{1}H NMR of QPD-OTf in acetone-*d6*

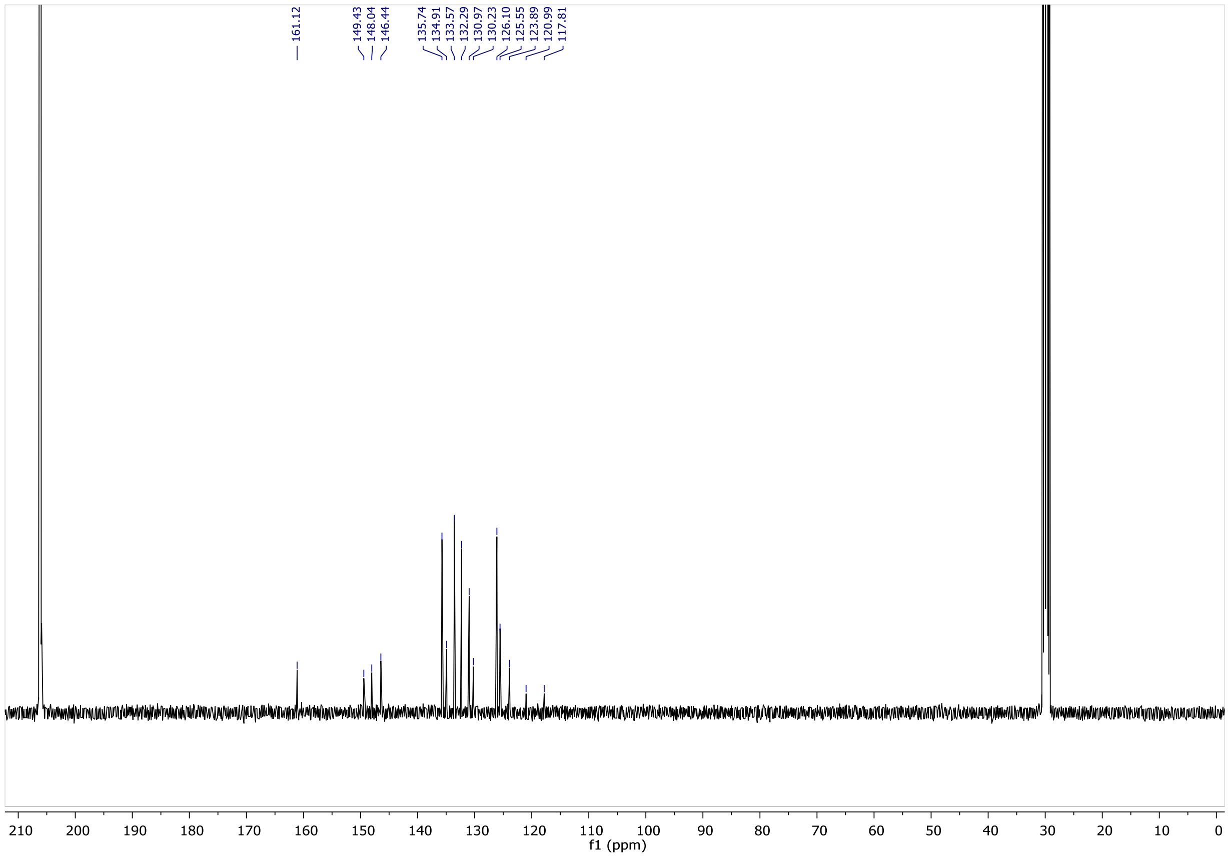

Supplementary Figure 22. ^{13}C NMR of QPD-OTf in acetone-*d6*

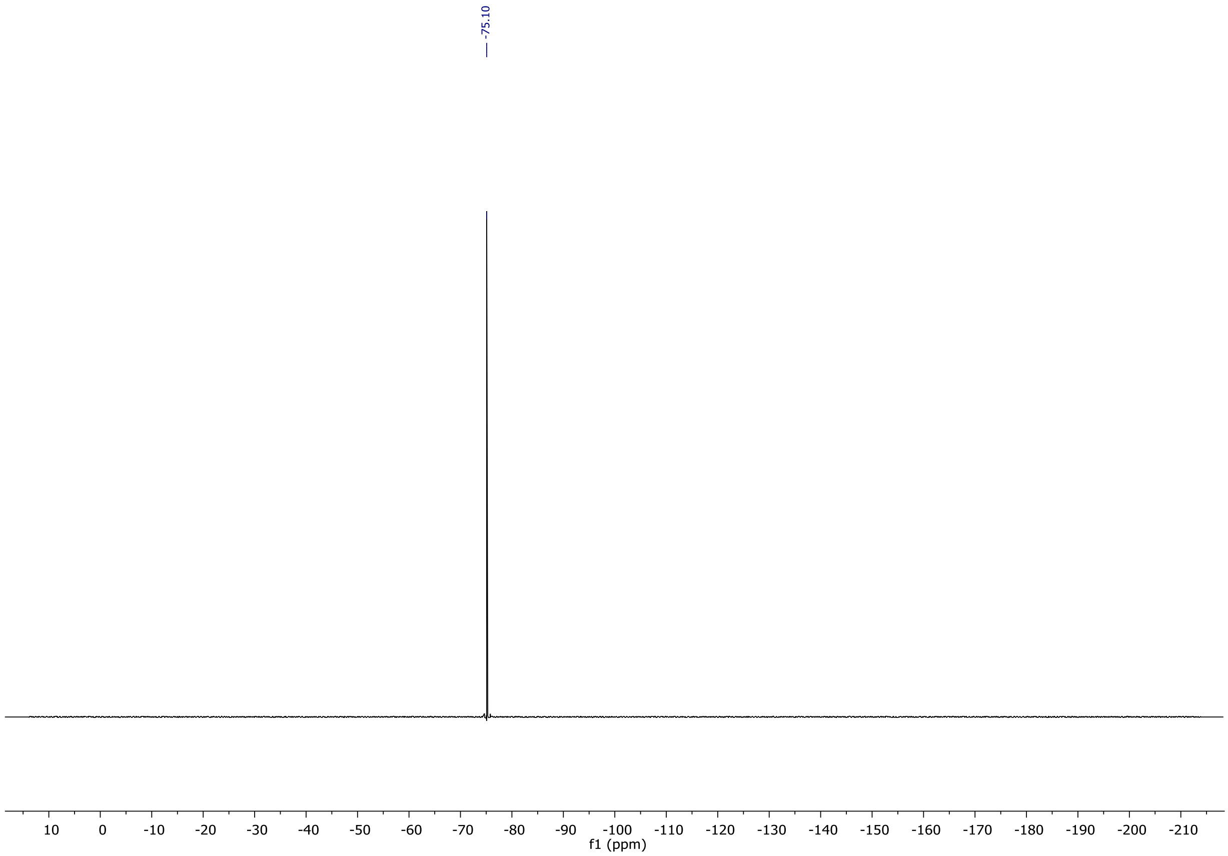

Supplementary Figure 23. ^{19}F NMR of QPD-OTf.

Supplementary Methods

Materials and methods

Chemical reactions were performed in anhydrous conditions under N_2. All reagents and solvents were purchased from commercial sources and were used without any further purification. Anhydrous solvents were obtained by passing them through commercially available alumina column (Innovative Technology, Inc., ® VA). Synthesized compounds were characterized using ^{1}H, ^{13}C, ^{19}F NMR, recorded on AVANCE 3 HD for 400 MHz using Acetone-d_6, as solvent, with residual solvent peaks (δ = 2.05 ppm; δ = 29.84 ppm). LC-MS spectra were recorded by using a DIONEX Ultimate 3000 UHPLC coupled with a Thermo LCQ Fleet Mass Spectrometer System (electrospray ionization (ESI)) operated in positive mode (condition for elution gradient: 0 min, A:B = 100:0; 4 min, A:B = 10:90; solution A: 0.01% aqueous TFA solution; solution B, 0.01 % TFA in HPLC grade acetonitrile; flow rate: 0.750 mL/min.). Transmitted light imaging was performed using an EVOS XL Core Imaging System with a 20X objective. Fluorescence imaging was carried out using a Leica SP8 with LIGHTNING module, a Zeiss LSM700 or a Zeiss LSM710 2P microscope with 63X objectives. All images were analyzed with Image J (Fiji). FIB-SEM rendering was performed with Imaris. pSF-mCherry-SNAP-Giantin plasmid was a kind gift of Howard Riezman's lab (University of Geneva, Switzerland). Docking calculations were performed with Autodock Vina. Data plotting was performed using GraphPad Prism8.

Synthesis of 4-chloro-2-formylphenyl trifluoromethanesulfonate

5-chloro-2-hydroxybenzaldehyde (300 mg, 1.92 mmol, 1 eq) was dissolved in dry DCM (5 mL) and cooled to 0 °C and trimethylamine (535 µL, 3.84 mmol, 2 eq) was added. After 5 min triflic anhydride (320 µL, 1.92 mmol, 1 eq) was added dropwise and the mixture was stirred at 0 °C for 2 hours. Then the mixture was diluted with H_2O (50 mL) and extracted with AcOEt (3x10 mL). The organic layers were combined, washed with brine, dried over Na_2SO_4 and concentrated under reduced pressure. The crude product was used in the next step without further purifications.

Synthesis of QPD-OTf.

4-chloro-2-formylphenyl trifluoromethanesulfonate (300 mg, 1.04 mg, 1 eq), 2-amino-5-chlorobenzamide (177 mg, 1.04 mmol, 1 eq), *p*-toluensulfonic acid (59 mg, 0.3 mmol, 0.3 eq) were suspended in EtOH (2.6 mL). The mixture was heated to reflux and stirred for 2 hours. The the mixure was cooled to 0 °C and 2,3-Dichloro-5,6-dicyano-1,4-benzoquinone (236 mg, 1.04 mmol, 1 eq) was added portion wise; the mixture turns to green, then to brown with a white precipitate. The suspension was diluted with cold EtOH (5 mL) and centrifuged. The white solid was washed twice with cold EtOH and dried under vacuum. The crude residue was purified by flash chromatography on silica gel (Pentane/EtOAc 9:1 to 6:4). Fractions containing the desired product were collected and dried under reduced pressure to afford QPD-OTf (360 mg, 80% yield).

LC-MS (ESI$^+$): RT= 2.92 min. m/z found: 438.94 $[M+H]^+$. HRMS (m/z): $[M+H]^+$ calcd. for $C_{15}H_8Cl_2F_3N_2O_4S^+$, 438.9528; found, 438.9534. ^{1}H NMR (400 MHz, Acetone-*d*$_6$): δ 11.69 (s, 1H), 8.18 (dd, *J* = 2.5, 0.5 Hz, 1H), 8.13 (d, *J* = 2.6 Hz, 1H), 7.89 (dd, *J* = 8.8, 2.5 Hz, 1H), 7.85 (dd, *J* = 8.9, 2.6 Hz, 1H), 7.82 (dd, *J* = 8.8, 0.5 Hz, 1H), 7.68 (d, *J* = 8.9 Hz, 1H). ^{13}C NMR (101 MHz, Acetone-*d*6): δ 161.12, 149.43, 148.04, 146.44, 135.74, 134.91, 133.57, 132.29, 130.97, 130.23, 126.10, 125.55, 123.89, 120.99, 117.81. ^{19}F NMR (282 MHz, Acetone-*d*6): δ -75.10. See Supplementary Fig. 20 for LC-MS traces and Supplementary Fig. 21, 22, 23 for ^{1}H, ^{13}C and ^{19}F NMR spectra.

QPD-OTf in Zymosan stimulated RAW246.7 cells

RAW246.7 were seeded into 3.5 cm glass bottom dishes with 10 mm microwell (Mattek); cells were incubated in DMEM medium at 37 °C under 5% CO_2 in a humidified incubator for 16 hours. Then media was removed, cells were washed twice with DPBS (Ca^{2+}, Mg^{2+}) and IgG-opsonized Zymosan A particles (20 µL) + QPD-OTf (20 µM) was added to cells in DMEM (-) (no additives). Cells were incubated at 37 °C under 5% CO_2 and imaged at different time points with a Zeiss LSM710 2P microscope.

SiR-Tubulin staining in QPD-OTf treated cells

U2OS cells ($2x10^5$) were seeded into 3.5 cm glass bottom dishes with 10 mm microwell (Mattek); cells were incubated in McCoy's 5A medium at 37 °C under 5% CO_2 in a humidified incubator for 16 hours. Then media was removed, cells were washed twice with DMEM (-) (no additives) and QPD-OTf (20 µM) was added to cells in DMEM (-) (no additives). Cells were incubated at 37 °C under 5% CO_2 for 4 hours. After washing twice with DMEM (-) (no additives), SiR-Tubulin (1 µM) was added and cells were incubated for 45 min at 37 °C under 5% CO_2. Cells were washed twice with DMEM (-) and imaged with a LEICA SP8 microscope.

Tubulin purification from bovine brain and tubulin labelling

Tubulin was purified from fresh bovine brain by two cycle of polymerisation and depolymerisation as previously described (Castoldi & Popov, 2003). A first polymerisation-depolymerisation cycle was performed in High-Molarity PIPES buffer [1 M PIPES-KOH at pH 6.9, 10 mM $MgCl_2$, 20 mM EGTA, 1.5 mM ATP and 0.5 mM GTP] supplemented with 1:1 glycerol and Depolymerisation buffer [50 mM MES-HCl at pH 6.6 and 1 mM $CaCl_2$] respectively. A second polymerisation-depolymerisation cycle was then performed: polymerisation in High-Molarity PIPES buffer and depolymerisation in 0.25XBRB80 complete after 15 min with 5XBRB80 to reach 1XBRB80 [80 mM PIPES at pH 6.8, 1 mM MgCl2 and 1 mM EGTA] respectively.

Labelled tubulin with ATTO-488, ATTO-565, or ATTO-647 (ATTO-TEC GmbH) and biotinylated tubulin were prepared as previously described (Hyman et al., 1991) with slight modification. Tubulin was polymerised in Glycerol PB solution [80 mM PIPES-KOH at pH 6.8, 5 mM $MgCl_2$, 1 mM EGTA, 1 mM GTP and 33 % (v/v) glycerol] for 30 min at 37°C and layered onto cushions of 0.1 M NaHEPES at pH 8.6, 1 mM $MgCl_2$, 1 mM EGTA and 60 % (v/v) glycerol followed by centrifugation. Pellet was resuspended in Resuspension buffer [0.1 M

NaHEPES at pH 8.6, 1 mM $MgCl_2$, 1 mM EGTA, 40% (v/v) glycerol] and incubated 10 min at 37°C with 1/10 volume of 100 mM ATTO-488, -565, or -647 NHS-fluorochrome or incubated 20 min at 37°C with 2 mM Biotin reagent. Labelled tubulin was sedimented onto cushions of BRB80 supplemented with 60 % glycerol, resuspended in BRB80, and a second polymerisation-depolymerisation cycle was performed before use. Labelling ratio was 13 % for ATTO-565.

rKin430-GFP expression and purification

All in vitro experiments were performed with truncated, EGFP-labelled kinesin-1 construct, rKin430-EGFP (referred as kinesin-1 in the text). The rKin430-GFP plasmid (a kind gift of Stefan Diez's laboratory) was expressed and purified as previously described (Rogers et al., 2001). *E.coli* BL21(DE3)[pLysS] expressing rKin430-GFP were lysed in a Lysis buffer [50 mM Na-Phosphate buffer at pH 7.5, 300 mM KCl, 10 % Glycerol, 1 mM $MgCl_2$, 20 mM ß-Mercaptoethanol, 0.2 mM ATP, 30 mM imidazole and protease inhibitors cocktail tablets (Roche)]. The cleared lysate was loaded into a pre-equilibrated HisTrap column (GE Healthcare 1mL HisTrap column) using an ÄKTA Pure Protein Purification System (GE Healthcare). After predefined washes, protein was eluted with Elution buffer [50 mM Na-Phosphate buffer at pH 7.5, 300 mM KCl, 10 % Glycerol, 1 mM $MgCl_2$, 0.2 mM ATP, 300 mM imidazole and 10 % (w/v) sucrose]. A dialysis was performed overnight to exchange the Elution buffer 20 mM NaHEPES at pH 7.7, 150 mM KCl, 1 mM $MgCl_2$, 0.05 mM ATP, 1 mM DTT and 20 % (w/v) Sucrose. Protein concentration was measured by Bradford method and the concentration was adjusted to 1.2 µg/µL using Centrifugal filter Amicon 30K (Millipore). Protein was aliquoted and stored in liquid nitrogen.

TIRF Imaging

Microscopy imaging was realized with an Axio Observer Inverted TIRF microscope (Zeiss, 3i) and a Prime BSI (Photometrics). A 100X objective (Zeiss, Plan-Apochromat 100X/1.46 oil DIC (UV) VIS-IR) were used. SlideBook 6 X64 software (version 6.0.17) was used to record time-lapse imaging. For in vitro, microscope stage conditions were controlled with the Chamlide Live Cell Instrument incubator (37 °C).

Flow chamber

Slides and coverslips were cleaned by two successive incubations and sonication: sonicated for 40 min in 1 M NaOH, rinsed in bidistillated water, sonicated in ethanol (96 %) for 30 min and rinsed in bidistillated water. Slides and coverslips were dried with an air gun, placed into a Plasma cleaner (Electronic Diener, Plasma surface technology) for plasma treatment, followed by 2 days incubation with tri-ethoxy-silane-PEG (Creative PEGWorks) or a 1:5 mix of tri-ethoxy-silane-PEG-biotin and tri-ethoxy-silane-PEG at 1 mg/ml in 96 % ethanol and 0.02 % HCl, with gentle agitation at room temperature. Slides and coverslips were then washed in ethanol (96 %), and bidistillated water, dried with air gun and stored at 4 °C. Flow chamber was assembled by fixing

with double tap a tri-ethoxy-silane-PEG treated slide with a 1:5 mix of tri-ethoxy-silane-PEG-biotin and tri-ethoxy-silane-PEG treated coverslip.

Microtubule seeds were prepared at 10 µM tubulin concentration (20 % ATTO-647-labelled tubulin and 80 % biotinylated tubulin) in BRB80 supplemented with 0.5 mM GMPCPP (Jena Bioscience) for 1 hour at 37°C. Seeds were incubated with 1 µM Paclitaxel (Sigma) for 45 min at 37 °C, centrifuged (50.000 rpm at 25°C for 15 min), resuspended in BRB80 supplemented with 1 µM Paclitaxel and 0.5 mM GMPCPP and stored in liquid nitrogen.

To observe precipitation of QPD in presence of stabilised microtubule seeds, we injected seeds in BRB80 supplemented with 20 µM QPD, 0.2 % BSA and anti-bleaching buffer (10 mM DTT, 0.3 mg/mL glucose, 0.1 mg/mL glucose oxidase, 0.02 mg/mL catalase, 0.125 % methyl cellulose (1500 cP, Sigma), 1 mM GTP, 2.7 mM $MgCl_2$ and 2.7 mM AMP-PMP), inside the flow chamber. No precipitation was observed after 2 hours under the microscope.

To study the precipitation of QPD in presence of dynamic microtubules we polymerised microtubules from seeds in BRB80 supplemented with 14 µm unlabelled tubulin, 20 µM QPD and 1 mM GTP at 37 °C in an Eppendorf-tube. For direct observation of precipitation, we polymerised microtubules from microtubule seeds in the flow chamber. The Flow chamber was prepared by injecting successively 50 µg/mL neutravidin (ThermoFisher), BRB80, GMPCPP microtubule seeds, and washed with BRB80 to remove unattached seeds. Microtubule polymerised from the seeds with 12 µM tubulin (20 % Atto-565 labelled) in BRB80 supplemented with an anti-bleaching buffer [10 mM DTT, 0.3 mg/mL glucose, 0.1 mg/mL glucose oxidase, 0.02 mg/mL catalase, 0.125 % methyl cellulose (1500 cP, Sigma), 1 mM GTP] and 0.2% BSA. The chamber was incubated for 15 min at 37 °C for polymerisation. The chamber sealed for imaging of microtubule dynamics and QPD precipitation at 37 °C of microtubule dynamics. Images were recorded every 3 min for 2 hours.

Molecular docking

Docking calculations were performed with Autodock Vina.[1] Receptor (PDB structure: 3J8Y for kinesin-1, 4AP0 for Eg5) and ligand preparation were performed in AutodockTools. Results were displayed with PyMol.

Supplementary References

1. O. Trott, A. J. Olson, AutoDock Vina: improving the speed and accuracy of docking with a new scoring function, efficient optimization and multithreading, Journal of Computational Chemistry 31 (2010) 455-461.
