# Supplementary material for: Kinesin-1 activity recorded in living cells with a precipitating dye
Source: Nat Commun. 2021 Mar 5;12:1463. doi: 10.1038/s41467-021-21626-1 (PMC7935933; doi:10.1038/s41467-021-21626-1)
Supplement: Supplementary file 2 — Reporting Summary [file 41467_2021_21626_MOESM2_ESM.pdf]

nature research

Corresponding author(s): Charlotte Aumeier, Nicolas Winssinger

Last updated by author(s): Jan 25, 2021

Reporting Summary

Nature Research wishes to improve the reproducibility of the work that we publish. This form provides structure for consistency and transparency in reporting. For further information on Nature Research policies, see our Editorial Policies and the Editorial Policy Checklist.

Statistics

For all statistical analyses, confirm that the following items are present in the figure legend, table legend, main text, or Methods section.

n/a	Confirmed	
☐	☒	The exact sample size (n) for each experimental group/condition, given as a discrete number and unit of measurement
☐	☒	A statement on whether measurements were taken from distinct samples or whether the same sample was measured repeatedly
☐	☒	The statistical test(s) used AND whether they are one- or two-sided<br>*Only common tests should be described solely by name; describe more complex techniques in the Methods section.*
☒	☐	A description of all covariates tested
☒	☐	A description of any assumptions or corrections, such as tests of normality and adjustment for multiple comparisons
☐	☒	A full description of the statistical parameters including central tendency (e.g. means) or other basic estimates (e.g. regression coefficient) AND variation (e.g. standard deviation) or associated estimates of uncertainty (e.g. confidence intervals)
☐	☒	For null hypothesis testing, the test statistic (e.g. F, t, r) with confidence intervals, effect sizes, degrees of freedom and P value noted<br>*Give P values as exact values whenever suitable.*
☒	☐	For Bayesian analysis, information on the choice of priors and Markov chain Monte Carlo settings
☒	☐	For hierarchical and complex designs, identification of the appropriate level for tests and full reporting of outcomes
☒	☐	Estimates of effect sizes (e.g. Cohen's d, Pearson's r), indicating how they were calculated

Our web collection on statistics for biologists contains articles on many of the points above.

Software and code

Policy information about availability of computer code

Data collection	No software were used
Data analysis	Autodock4 Vina, Autodock Tools 1.5.6, PyMOL2, Atlas5 software (Fibics), ImageJ (Fiji) 1.53c, Imaris 9.3.1, GraphPad Prism 8.

For manuscripts utilizing custom algorithms or software that are central to the research but not yet described in published literature, software must be made available to editors and reviewers. We strongly encourage code deposition in a community repository (e.g. GitHub). See the Nature Research guidelines for submitting code & software for further information.

Data

Policy information about availability of data

All manuscripts must include a data availability statement. This statement should provide the following information, where applicable:

- Accession codes, unique identifiers, or web links for publicly available datasets
- A list of figures that have associated raw data
- A description of any restrictions on data availability

The authors declare that all data supporting the findings of this study are available within the article and its supplementary information files. The datasets generated during and/or analysed during the current study, together with the source data have been deposited in the Zenodo repository and are available at DOI: 10.5281/zenodo.4461867.

Field-specific reporting

Please select the one below that is the best fit for your research. If you are not sure, read the appropriate sections before making your selection.

- [x] Life sciences
- [ ] Behavioural & social sciences
- [ ] Ecological, evolutionary & environmental sciences

For a reference copy of the document with all sections, see nature.com/documents/nr-reporting-summary-flat.pdf

Life sciences study design

All studies must disclose on these points even when the disclosure is negative.

Sample size	Sample size was chosen case by case to account for representative examples of the behavior observed. For quantitative experiments based on cell imaging sample size is >10.
Data exclusions	Data were not excluded.
Replication	Experiments were replicated at least two times; all attempts of replication were successful.
Randomization	No trials that require randomization were used within the study.
Blinding	Results acquired were not predictable a priori, therefore blinding was not employed for this study.

Reporting for specific materials, systems and methods

We require information from authors about some types of materials, experimental systems and methods used in many studies. Here, indicate whether each material, system or method listed is relevant to your study. If you are not sure if a list item applies to your research, read the appropriate section before selecting a response.

Materials & experimental systems

n/a	Involved in the study	
☐	☒	Antibodies
☐	☒	Eukaryotic cell lines
☒	☐	Palaeontology and archaeology
☒	☐	Animals and other organisms
☒	☐	Human research participants
☒	☐	Clinical data
☒	☐	Dual use research of concern

Methods

n/a	Involved in the study	
☒	☐	ChIP-seq
☒	☐	Flow cytometry
☒	☐	MRI-based neuroimaging

Antibodies

Antibodies used	DM1-alpha raised in mouse (T6199), anti-mouse ALEXA-488, anti-rabbit ALEXA-568. anti-alpha-tubulin (Sigma, T6074); anti-UKHC (Santa Cruz Biotechnology, SC-133184), Rabbit anti-Mouse IgG, IgM (H+L) HRP Secondary Antibody (Invitrogen, 31457).
Validation	Antibodies are commercially available from Sigma-Aldrich, Santa Cruz Biotechnology and Thermo Scientific; antibodies are validated by the commercial source via Independent Antibody Verification.

Eukaryotic cell lines

Policy information about cell lines

Cell line source(s)	HeLa, U2OS, HEK293, MCF-7, RAW246.7 cells were purchased from ATCC; GFP-Tubulin Ptk2 cells was a kind gift from Franck Perez (originally purchased from CLONTECH Laboratories, Inc.). GFP-Tubulin CRISPR knock-in Hela cells were generated by Charlotte Aumeier.
Authentication	Authentication by microscopy.
Mycoplasma contamination	Cell were tested for mycoplasma contamination routinely by staining with Hoechst 33342 and always resulted not contaminated.
Commonly misidentified lines (See ICLAC register)	No commonly misidentified cells lines were used within this study.
