# Supplementary material for: Kinesin-1 activity recorded in living cells with a precipitating dye
Source: Nat Commun. 2021 Mar 5;12:1463. doi: 10.1038/s41467-021-21626-1 (PMC7935933; doi:10.1038/s41467-021-21626-1)
Supplement: Supplementary file 4 — Description of Additional Supplementary Files [file 41467_2021_21626_MOESM4_ESM.pdf]

**Description of Additional Supplementary Files**

**File**: Supplementary Movie 1

**Description**: crystals growth over time in live PTK2 cells treated with 20 µM QPD-OTf.

**File**: Supplementary Movie 2

**Description**: HeLa cells treated with QPD-OTf (20 µM, 1.5 hours), washed and imaged over time to observe crystal disappearance.

**File**: Supplementary Movie 3

**Description**: crystals growth from Golgi apparatus in U2OS cells transfected with mCherry-Giantin and treated with 20 µM QPD-OTf (magenta: Golgi; cyan: crystal).
